# *Yellow-Leaf 1* encodes a magnesium-protoporphyrin IX monomethyl ester cyclase, involved in chlorophyll biosynthesis in rice (*Oryza sativa L*.)

**DOI:** 10.1371/journal.pone.0177989

**Published:** 2017-05-30

**Authors:** Zhonghua Sheng, Yusong Lv, Wei Li, Rongjian Luo, Xiangjin Wei, Lihong Xie, Guiai Jiao, Gaoneng Shao, Jianlong Wang, Shaoqing Tang, Peisong Hu

**Affiliations:** 1 State Key Laboratory of Rice Biology, Key Laboratory of Rice Biology and Breeding of Ministry of Agriculture, China National Rice Research Institute, Hangzhou, China; 2 National Key Laboratory of Crop Genetic Improvement, Huazhong Agricultural University, Wuhan, China; 3 Agricultural College of Hunan Agricultural University, Changsha, China; University of Western Sydney, AUSTRALIA

## Abstract

Magnesium-protoporphyrin IX monomethyl ester cyclase (MPEC) catalyzes the conversion of MPME to divinyl protochlorophyllide (DVpchlide). This is an essential enzyme during chlorophyll (Chl) biosynthesis but details of its function in rice are still lacking. Here, we identified a novel rice mutant *yellow-leaf 1* (*yl-1*), which showed decreased Chl accumulation, abnormal chloroplast ultrastructure and attenuated photosynthetic activity. Map-based cloning and over-expression analysis suggested that *YL-1* encodes a subunit of MPEC. The YL-1 protein localizes in chloroplasts, and it is mainly expressed in green tissues, with greatest abundance in leaves and young panicles. Results of qRT-PCR showed that Chl biosynthesis upstream genes were highly expressed in the *yl-1* mutant, while downstream genes were compromised, indicating that *YL-1* plays a pivotal role in the Chl biosynthesis. Furthermore, the expression levels of photosynthesis and chloroplast development genes were also affected. RNA-seq results futher proved that numerous membrane-associated genes, including many plastid membrane-associated genes, have altered expression pattern in the *yl-1* mutant, implying that *YL-1* is required for plastid membrane stability. Thus, our study confirms a putative MPME cyclase as a novel key enzyme essential for Chl biosynthesis and chloroplast membrane stability in rice.

## Introduction

Chloroplasts are specific organelles in higher plants and the site of Chl biosynthesis. Chl is extremely important in photosynthesis because it plays an essential role in harvesting light energy and converting it to chemical energy [1]. Improving the efficiency of Chl biosynthesis is an excellent way to drive the accumulation of more photoassimilates and ultimately increase crop yield [2, 3]. Hence, breeders strive to develop plants with a long stay-green period in order to increase the yield potential.

The Chl biosynthesis pathway is complex, requiring many essential enzymes and other protein regulators. 5-Aminolaevulinic acid (ALA) is the precursor of chlorophyll and other tetrapyrrole end products [4]. Currently, at least 15 enzymes have been identified as involved in the ultimate conversion of ALA to Chl a and b [5]. Glutamyl-tRNA synthetase (GluRS) is the initial enzyme that regulates the formation of ALA [6, 7] and mutation of OsGluRS caused yellow leaf and dwarf height phenotypes in rice [7]. Magnesium ion chelating enzyme (Mg-chelatase) is the second rate-limiting enzyme in chlorophyll synthesis, and catalyzes formation of the magnesium-protoporphyrin IX complex from magnesium ions and protoporphyrin IX [8, 9]. ChlD, ChlH and ChlI are the main subunits of Mg-chelatase. T-DNA insertion into *OsChlH* caused impediment of Chl biosynthesis and subsequent death of the plants [10]. Divinyl reductase (DVR) converts 8-vinyl groups on various chlorophyll intermediates to ethyl groups, and is indispensable for Chl biosynthesis [11–13]. The *OsDVR* gene mutant exhibited a yellow-green leaf phenotype, reduced Chl level, arrested chloroplast development, and retarded growth rate [13]. In the last step of Chl biosynthesis, Chl *b* is synthesized from Chl *a* through the oxidation of a methyl group on the D ring, which is catalyzed by chlorophyllide a oxygenase (CAO) [14–16]. Two homologous genes of *CAO*, *OsCAO1* and *OsCAO2*, have been identified from the rice genome. *OsCAO1* plays a major role in Chl b synthesis and chloroplast development under the light, whereas *OsCAO2* may function in the dark. A study using T-DNA mutants found that *OsCAO2* knockout mutant leaves do not differ significantly from wild-type leaves, whereas *OsCAO1* knockout mutants had pale green leaves [17]. Chlorophyll (Chl) synthase catalyzes esterification of chlorophyllide to complete the last step of Chl biosynthesis [18, 19]. *YGL1* is the main Chl synthase gene which has been cloned. The *ygl1* mutant shows yellow-green leaves in young plants, with decreased Chl synthesis, increased level of tetrapyrrole intermediates, and delayed chloroplast development [20].

Most of enzymes inovlved in Chl biosynthesis in rice have been identified and characterized from a series of Chl-deficient mutants. One of the least understood enzymatic steps is formation of the homocyclic ring, which is a characteristic feature of all chlorophyll molecules, including those found in bacteria. In chloroplasts, formation of the isocyclic ring is an aerobic reaction catalyzed by MPEC[4], which controls the conversion of MPME to divinyl protochlorophyllide (DVpchlide). This pathway has still not been elucidated in rice. MPEC is a highly conserved protein in bacteria, green algae and plants, and was originally identified as the *acsF* gene in *Rubrivivax gelatinosus*, a photosynthetic bacterium [21]. In algae *copper response defect 1* (*Crd1*) mutants, homologs of *AcsF*, encoding the catalytic component were identified, which were required for the accumulation of Photosystem I (PSI) and light-harvesting complex I (LHCI) during hypoxia or copper deficiency [22, 23]. In higher plants, a protein encoded by *PNZIP* was the first isolated catalytic subunit of the MPEC, and contains a leucine zipper domain [24]. PNZIP in *Pharbitis nil* has been found to be regulated by phytochrome and circadian rhythms [24, 25]. *Xantha-l* encodes a membrane-bound cyclase subunit in barley. The *xantha-l* mutants are leaky and able to synthesize a limited amount of chlorophyll, which gives them a pale green color. They supports the view that the aerobic cyclase is most likely composed of the products of three genes, a souble protein, a membrane-bound component encoded by *Xantha-l*, and another membrane-bound component encoded by *Viridis-k* [26]. Ycf54 has been identified as a potential componet of MPEC, stimulating its activity. Ycf54 is likely to be a membrane-bound component different from the *Viridis-k* and the *Xantha/AcsF* gene products [27]. In addition, the homology of *YL-1* in *Arabidopsis*, *CHL27* has been identified as required for the synthesis of protochlorophyllide a. The *CHL27* mutant has a chlorotic phenotype with the accumulation of MPEC as a cyclase reaction substrate [28, 29]. Recently, a negative regulator of tetrapyrrole biosynthesis (FLU) NADPH: protochlorophyllide oxidoreductase (POR), and CHL27 were found to form a FLU-CHL27-POR cyclase complex, which is requred for substrate channeling from MgPME to Chlide and also ALA synthesis in chloroplasts [30]. Despite extensive investigations in many species, the function of MPEC in rice, one of main crops in the world, is still not fully elucidated. The photosynthesis and chloroplast morphology phenotypes conferred by mutations in MPEC have mostly not been reported, although recently, *ygl8*, a novel MPEC defect mutant in rice, has been identified. This also shows a chlorisis phenotype with faults in Chl synthesis [31].

In this study, we identified a novel Chl-defects mutant, named yellow leaf 1 (*yl-1*), of which the seedling leaves and panicles are deficient in Chl content, corresponding to its chlorisis phenotype through the whole developmental stages. It was shown that *YL-1* encodes a subunit of MPEC, which play important roles in conversion of MPME to divinyl Dvpchlide.Transcription levels of many genes associated with the Chl biosynthesis, chloroplast development and photosynthesis were significantly changed in the *yl-1* mutant, compared to wild-type. These results indicated that the function of YL-1 in MPEC complex is essential for normal Chl biosynthesis and which would used for roguing in hybrid rice production.

## Materials and methods

### Plant materials and planted conditions

The *yl-1* mutant was identified from EMS-induced mutant library of *indica* super early-season rice cv. Zhongjiazao 17. An F_2_ population derived from the cross between *yl-1* and tropic *japonica* cv. D50 was used for genetic analysis and gene mapping. The *yl-1* and wild-type plants were grown in paddy fields during normal growing seasons at the China National Rice Research Institute, in Hangzhou (30°N latitude), as well as in a growth chamber (12/12h light/dark; light intensity 300 μmol m^−2^ s^−1^) at a constant temperature of either 30, 26 or 22°C. The F_2_ and transgenic plants were grown in the Hangzhou paddy fields.

### Chlorophyll content and transmission electron microscopy analysis

Leaf chlorophyll contents were determined according to the method described in [20]. A 0.2g sample of fresh leaf was immersed in 10 mL ethanol for 48 h in the dark, following which plant debris was removed by centrifugation and the supernatant analyzed by spectrophotometric scanning at 665, 649 and 470 nm. Chloroplast structures were examined in the third leaf of both *yl-1* and wild-type cv. Zhongjiazao 17 plants using transmission electron microscopy (TEM) as described [32] with minor modifications. Briefly, Samples of wild-type and *yl-1* mutant leaves were prepared for transmission electron microscopy by cutting into small pieces, fixed in 2.5% glutaraldehyde in a phosphate buffer at 4°C for 4 h, rinsed and incubated overnight in 1% OsO_4_ at 4°C, dehydrated through an ethanol series, and infiltrated with a graded series of epoxy resin, and then embedded in Epon 812 resin. Thin sections were obtained using a diamond knife and a Reichert OM_2_ ultramicrotome, stained in 2% uranyl acetate, and 10 mM lead citrate, pH 12, and then viewed with a Hitachi H-7650 transmission electron microscope.

### Photosynthetic rate and chlorophyll fluorescence analysis

An Li-6400 portable photosynthetic apparatus was used on a sunny day between 9~11 a.m, to determine the net photosynthetic rate (Pn) of wild-type and mutant plants at the beginning of the tillering stage. The red and blue light source were used for determination of photosynthetic rates of three representative leaves of both mutant and wild-type. The light quantum density was 1200 μmol m^-2^ s^-1^, velocity 500 μmol s^-1^. Chlorophyll fluorescence analysis of the third leaf at the 3-leaf stage were performed with a PAM-2000 portable chlorophyll fluorometer (MINIPAM; Walz; http://walz.com).

### Mapping and cloning of *YL-1*

Initially, 36 yellow plants of *yl-1*/D50 F_2_ progeny were used for coarse linkage analysis, and subsequently 1220 homozygous mutant F_2_ segregants were used to fine mapping of *yl-1*. The microsatellite and In-Del markers used to genotype these progeny were given in Electronic Supplementary Material (ESM) S3 Table. The positional cloning strategy were according to [33]. The total volume of the PCR reaction was 10μL, containing 1μL of 10×PCR buffer (25 mM/L MgCl_2_), 0.1 mM of dNTPs, 0.1 mM of each primer, 0.25μL of Taq DNA polymerase (2 U/μL), 1μL of genomic DNA and 4.95μL of dd H_2_O. The amplification protocol included an initial denaturation at 95°C for 3 min, 35 cycles of 45 s at 95°C, 30 s at 55°C and 45 s at 72°C, and a final extension step at 72°C for 8 min. PCR products less than 300 bp were separated by 6% native polyacrylamide gel and silver-stained for visualization. Finally, the genetype of each plant was identified.

### Vector construction and rice transformation

The full-length coding region of *YL-1* was isolated from Zhongjiazao 17 by PCR (primers sequences were listed in S3 Table), and then subcloned into the binary vector *pCAMBIA1305*::*Ubiqutin*:*GFP* under the control of the rice *Ubiqutin 1* promoter. The resultant plasmid was introduced into Agrobacterium tumefaciens strain EHA105, and then was introduced into *yl-1* mutant by means of Agrobacterium mediated transformation. The genotypes of transgenic plants were determined by the Neomycin phosphotransferase II (nptII) resistance detection and the phenotype analysis of transgenic lines.

### RNA extraction and qRT-PCR analysis

RNA of various organs harvested from *yl-1* and wild-type plants were isolated using a RNA extraction kit (Beijing Dingguo Biotechnology Co. Ltd.; http://www.dingguo.com), following the manufacturer’s instructions. The cDNA first strand was reverse-transcribed by using oligo (dT) as primer. The transcriptions of *YL-1* and a series of chlorophyll synthesis- and photosynthesis-associated genes were assessed using quantitative real-time PCR (qRT-PCR), with the rice *ubiquitin* gene (GenBank accession AF184280) as the control. The relevant primer sequences are listed in S3 Table.

### Subcellular localization of YL-1

The coding sequence of *YL-1* was amplified by PCR using the specific primer pairs shown in S3 Table. The PCR products were cloned into the pAN580-GFP vector between the cauliflower mosaic virus 35S promoter and the NOS terminator to form translational fusions with the N terminus of GFP. All transient expression constructs were separately transformed into rice protoplasts and incubated in the dark at 28°C for 16 h before examination according to the protocols described previously[34, 35]. The fluorescence of GFP was observed using a confocal laser scanning microscope (Leica TCS SP5).

### RNA-sequencing analysis

4-week-old leaves of both *yl-1* and wild type were sampled, and total RNA was extracted using Trizol reagent (Invitrogen, CA, USA) following the manufacturer's procedure. The total RNA quantity and purity were analyzed by Bioanalyzer 2100 and RNA 6000 Nano LabChip Kit (Agilent, CA, USA) with RIN number >7.0. RNA-sequencing analysis was then performed on an Illumina Hiseq2000/2500 (LC Sciences, USA) following the vendor's recommended protocol.

## Results

### The *yl-1* mutant shows a yellow leaf and panicle phenotype with reduced Chl accumulation through the whole growth phases

**Fig 1:**
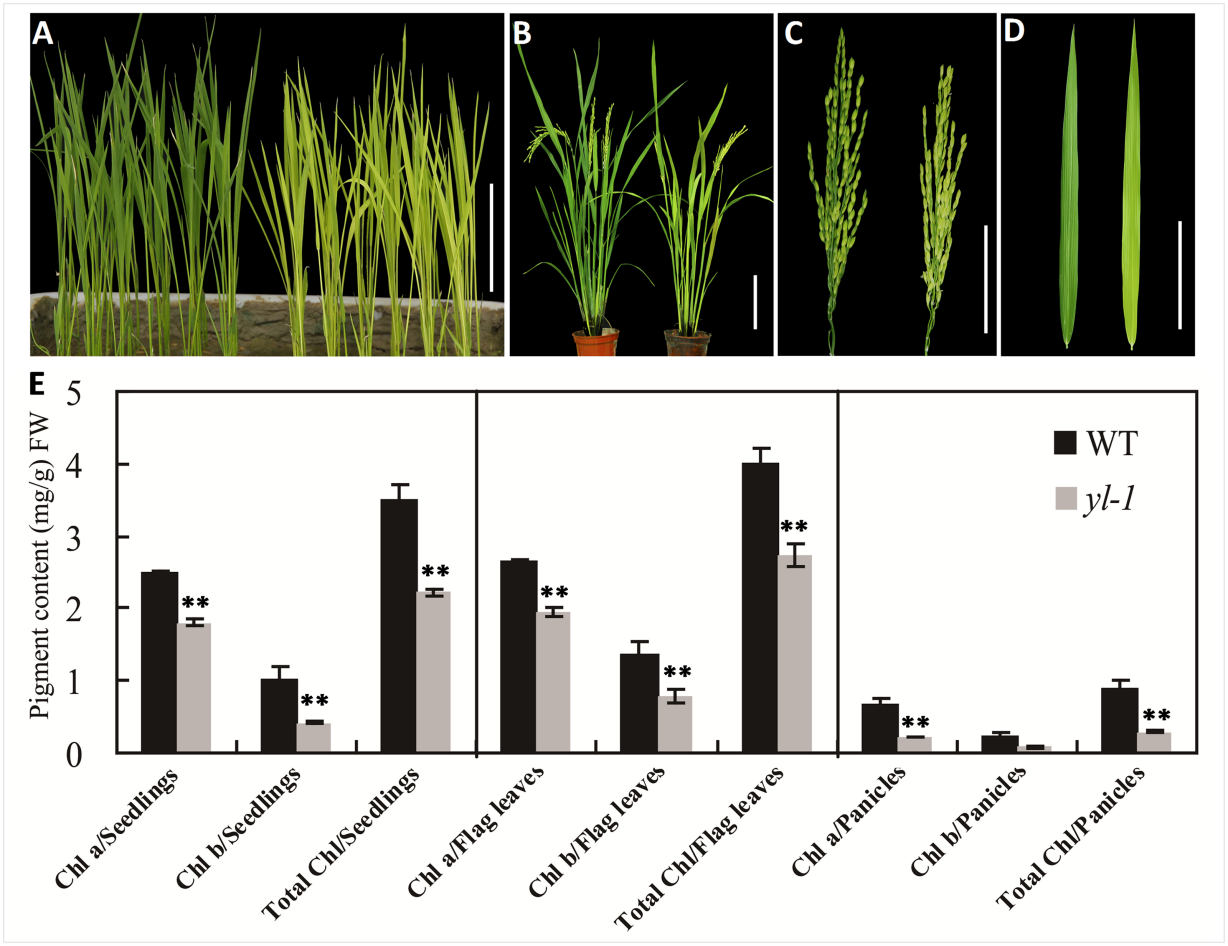
Phenotypic characterization of wild-type (WT) and *yl-1* mutants. **A**, Phenotypes of 3-week-old wild-type and *yl-1* seedlings (from left to right), bar = 10 cm. **B**, Phenotypes of wild-type and *yl-1* at the heading stage grown in paddy field, bar = 20 cm. **C-D**, Enlarged views of spikelets (**C**) and flag leaves (**D**) of wild-type and *yl-1* in **B**, bars = 5 cm. **E**, Chlorophyll content of leaves in wild-type and *yl-1* mutants at three different developmental stages: the seedlings stage; flag leaves and young panicles of the heading stage. Data are means ± SD (n = 5). Chl.a, Chlorophyll a; Chl.b, chlorophyll b; FW, fresh weight. The asterisks indicate statistical significance between the wild-type and the mutants, as determined by the Student’s *t*–test (* *P* < 0.05; ** *P* < 0.01).

**Fig 2:**
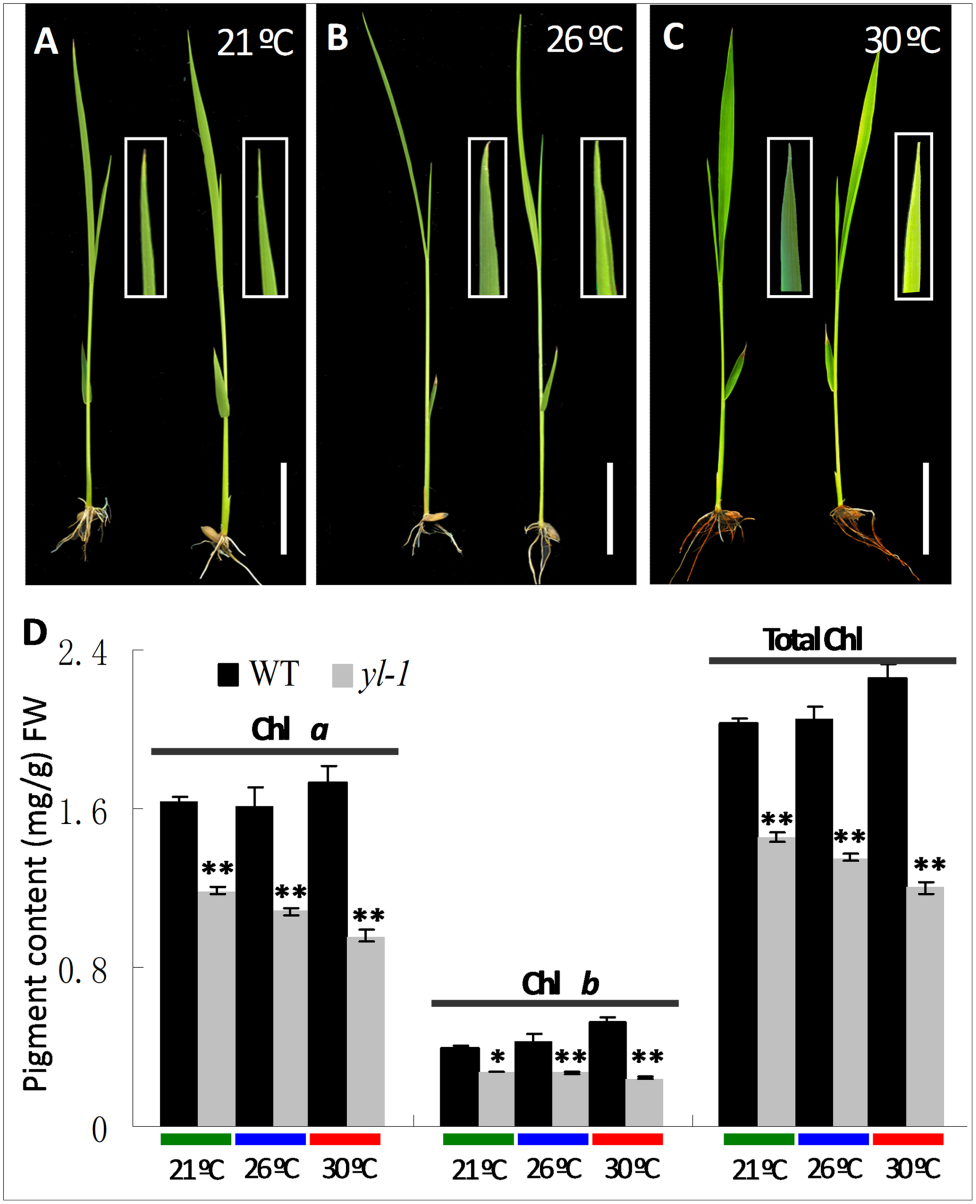
Chlorophyll content of leaves in wild-type and *yl-1* mutant at three different temperatures. **A-C**, 10-day-old seedlings of wild-type and *yl-1* mutant at 21°C (**A**), 26°C (**B**) and 30°C (**C**); bars = 2 cm. **D**, Chlorophyll content of leaves in wild-type and *yl-1* mutant at 21°C (**A**), 26°C (**B**) and 30°C (**C**). Data are means ± SD (n = 5). The asterisks indicate statistical significance between the wild-type and the mutants, as determined by the Student’s *t*–test (* *P* < 0.05; ** *P* < 0.01).

We measured Chl a, Chl b and total Chl accumulation in the leaves and young panicles of both *yl-1* mutant and wild-type plants at different developmental stages. The former exhibited a chlorosis leaf and yellow panicle phenotype throughout all developmental stages (Fig 1A–1D). Significantly reduced levels of Chl were detected in *yl-1*, with 30%, 35% and 60% reductions in Chl a and 65%, 50% and 70% reductions in Chl b of 4-week-old leaves, flag leaves and young panicles, respectively (Fig 1E). Furthermore, compared to the wild-type, the *yl-1* mutant exhibited significantly reduced plant height, grains per panicles and grain yield per plant, but with increased tiller number and seed setting rate (S1 Fig). Taken together, this suggests that the *yl-1* mutant phenotype results from reduced rates of Chl accumulation. To further identify whether the yellow leaf phenotype of *yl-1* is affected by external temperature changes, we analysed the Chl a, Chl b and total Chl content at 21°C, 26°C and 30°C. Our results showed that *yl-1* exhibited the yellow leaf phenotype under each of the three different temperature conditions and Chl contents were always much lower than those of the wild-type (Fig 2). However, Chl accumulation was much lower at 30°C than at 21°C, and also a more obviously chlorotic phenotype was observed at 30°C (Fig 2), which indicates that the *yl-1* mutant is intolerant of higher temperature environments.

### The *yl-1* mutant presents abnormal chloroplast development and attenuated photosynthetic efficiency

**Fig 3:**
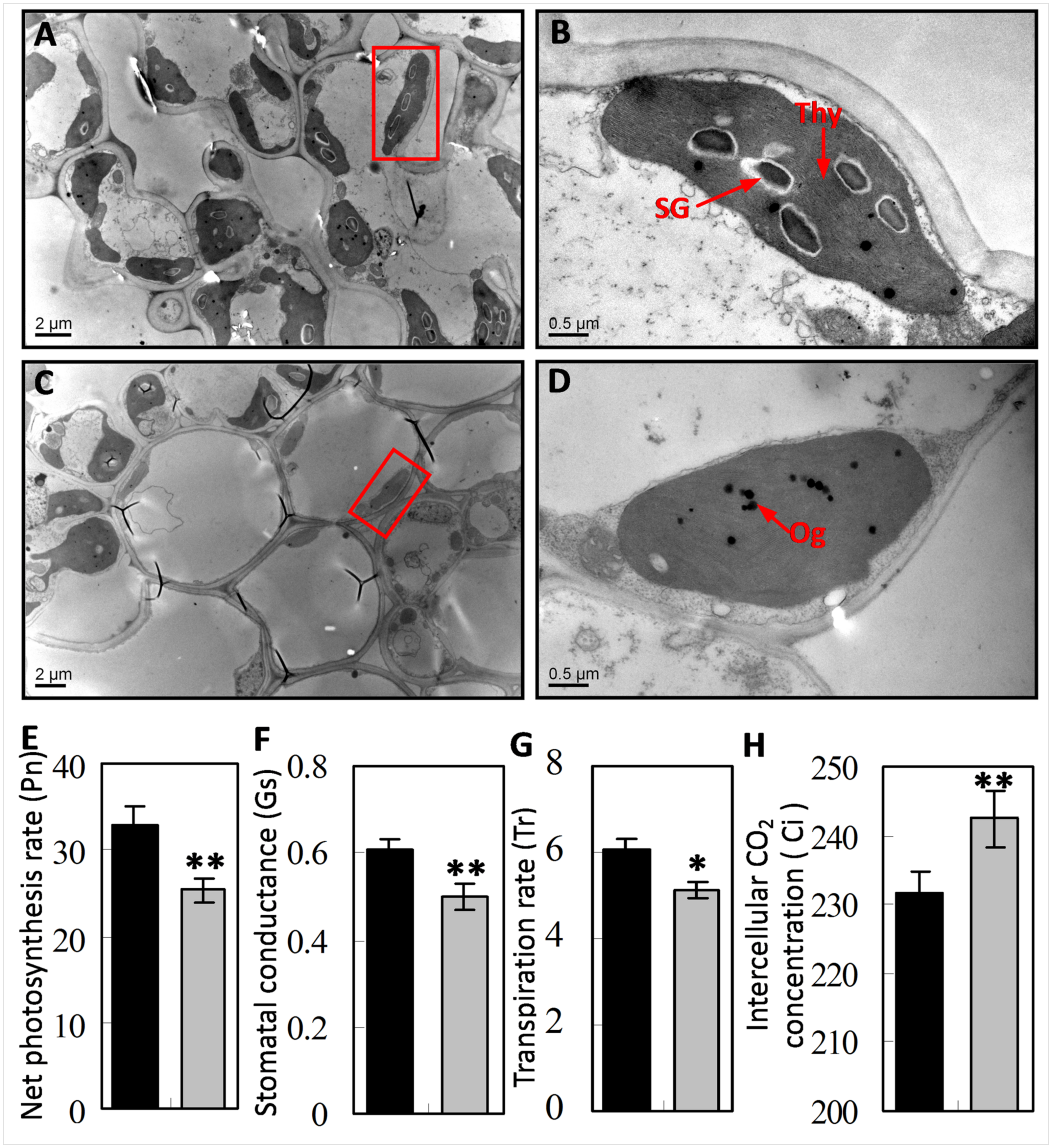
Chloroplast ultrastructures and photosynthetic parameters in wild-type and *yl1-1* mutant. **A-D**, Transmission electron microscopy (TEM) of chloroplasts from Leaf 4 of the wild-type (**A-B**) and *yl-1* mutant (**C-D**) seedlings at 3-week-old. **E-H**, photosynthetic parameters in wild-type (left) and *yl1-1* mutant (right). Data are presented as means ± SD (n = 5). *Thy* thylakoid lamellar; *SG* starch granule; *OG* osmiophilic plastoglobuli. Asterisk indicates significant difference between the wild-type and *yl-1* mutant. The asterisks indicate statistical significance between the wild-type and the mutants, as determined by the Student’s *t*–test (* *P* < 0.05; ** *P* < 0.01).

To investigate whether the lack of chlorophyll synthesis in the *y1-1* mutant was accompanied by defective chloroplast development, we compared the ultrastructure of plastids between *yl-1* mutant and wild-type plants using transmission electron microscopy (TEM). Chloroplast ultrastructures were observed in the 3^rd^ leaves of L4 seedlings. The results revealed that chloroplasts in the leaves of wild-type plants displayed well-developed membrane structure, with dense thylakoids arranged in the grana and in the membranes interconnecting of grana (Fig 3A and 3B). However, granas stacked in the *yl-1* mutant appear less dense and with many osmiophilic plastoglobuli. Thus, unlike the wild-type, leaves of young *yl-1* mutant plants display characteristics of ongoing cell senescence (Fig 3C and 3D).

The photosynthetic parameters of *yl-1* and wild-type plants grown in the field at the heading stage were measured. As expected, significantly reduced net photosynthetic rate (Pn), stomatal conductance (Gs) and transpiration rate (Tr) and higher intercellular CO_2_ concentration (Ci), were detected in *yl-1* compared to wild-type plants (Fig 3E–3H). To test whether the photosynthetic apparatus was affected in *yl-1*, some key light-induced chlorophyll fluorescence parameters of PSI and PSII were compared in *yl-1* and wild-type plants. Distinct decreases in the electron transport rate (ETR), photochemicalquenching (Qp) and maximal efficiency of PSII photochemistry (*Fv/Fm*) were oberserved in the *yl-1* mutant (Table 1). These observations indicated that the *yl-1* mutant suffer from defects affecting photosynthesis.

**Table 1:** Main photosynthetic parameters of wild-type and *yl-1* mutant, The asterisks indicated statistical significance between the wild-type and *yl-1*, as determined by the Student’s *t*–test (* *P* < 0.05; ** *P* < 0.01).

	*Fm*	*Fo*	*Fv/Fm*	*NPQ*	*Qp*	*ETR*
**WT**	0.33±0.02	0.10±0.006	0.70±0.04	0.26±0.03	0.74±0.02	63.93±1.97
***yl-1***	0.21±0.03**	0.11±0.01	0.48±0.02**	0.27±0.04	0.55±0.01**	55.38±1.29**

### Map-based cloning of *YL-1*

**Fig 4:**
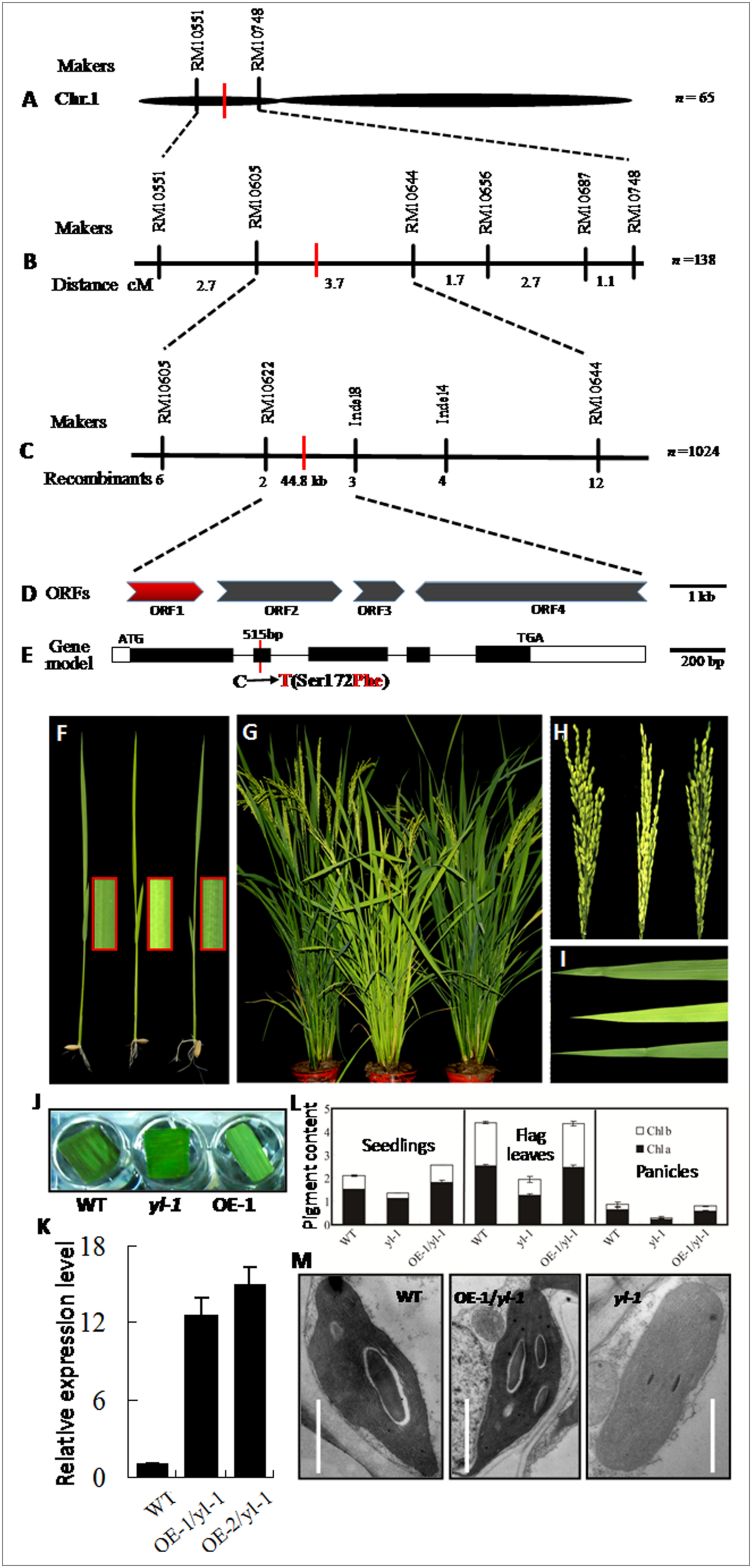
Map-based cloning of *YL-1* gene. **A-B**, Position of the *YL-1* locus in an 3.7 cM interval on Chr. 1 between SSR markers RM10605 and RM10644. **C**, *YL-1* was narrowed to a 44.8 kb region between the SSR marker RM10622 and Indel marker Indel 18. **D**, 4 putative ORFs in the 44.8 kb genomic region. **D**, Gene structure of the *YL-1* candidate gene (ORF1). The black boxes represent exons, the lines between them represent introns, and the white boxes means 5,UTR and 3,UTR of *YL-1*. **F-I**, Phenotype of wild-type, *yl-1* and T_1_ plant of transgenic *yl-1*expressing *YL-1* at the seedling (**F**) and heading (**G-I**) stages. Hygromycin resistance detection (**J**) and qRT-PCR of *YL-1* (**K**). **L-M**, Chlorophyll content (**L**) and chloroplast ultrastructures (**M**) in cells of third leaf of wild-type, *yl-1* and T_1_ transgenic plants expressing *YL-1*. Data in (**K**, **L**) are shown as means ± SD from 5 individual replicates.

The F_1_ young leaves of *yl-1*×cv. D50 were similar to those of wild-type cv.Zhongjiazao17, while the segregation behavior in the derived F_2_ population was consistent with the Mendelian monogenic ratio of three wild-type phenotype to one chlorisis phenotype (S1 Table). Linkage analysis fixed the location of the *yl-1* into a 3.7-cM interval on chromosome 1, between the SSR markers RM10605 and RM10644 (Fig 4A and 4B). Using 1220 mutation phenotype plants from the F_2_ population derived from *yl-1* × cv. D50, the location of *yl-1* was then narrowed down to a 44.8 kb physical region between SSR marker RM10622 and Indel marker Indel8 (Fig 4C). Within this region, 4 open reading frames (ORFs) were predicted (http://www.gramene.org/; Fig 4D). Genomic sequencing analysis of the 4 ORFs revealed that the first ORF (*LOC_Os01g17170*) carries a single-nucleotide transition (C→T) at the position 515 bp from the start codon ATG in *yl-1* (Fig 4E), leading to the transition of an amino acid residue from serine (Ser) in wild-type to phenylalanine (Phe) in *yl-1* (Fig 4E). Thus, *ORF1* was the candidate gene of *yl-1*.

To verify the identity of *yl-1*, the plasmid *Ubi*:*YL-1* containing wild-type *YL-1* cDNA region driven by the maize *Ubiquitin* promoter was transformed into *yl-1* via the *Agrobacterium*-mediated method. As expected, the yellow-leaf seedlings, flag leaves and panicles of *yl-1* containing *YL-1* cDNA recovered to a normal green phenotype (Fig 4F–4K). Moreover, chlorophyll content, chloroplast ultrastructural structure and the relative expression of *YL-1* in the transgenic positive lines returned to normal levels (Fig 4L and 4M). Thus, *ORF1* (*LOC_Os01g17170*) indisputably corresponds to *YL-1*.

### *YL-1* encodes an MgPME cyclase

**Fig 5:**
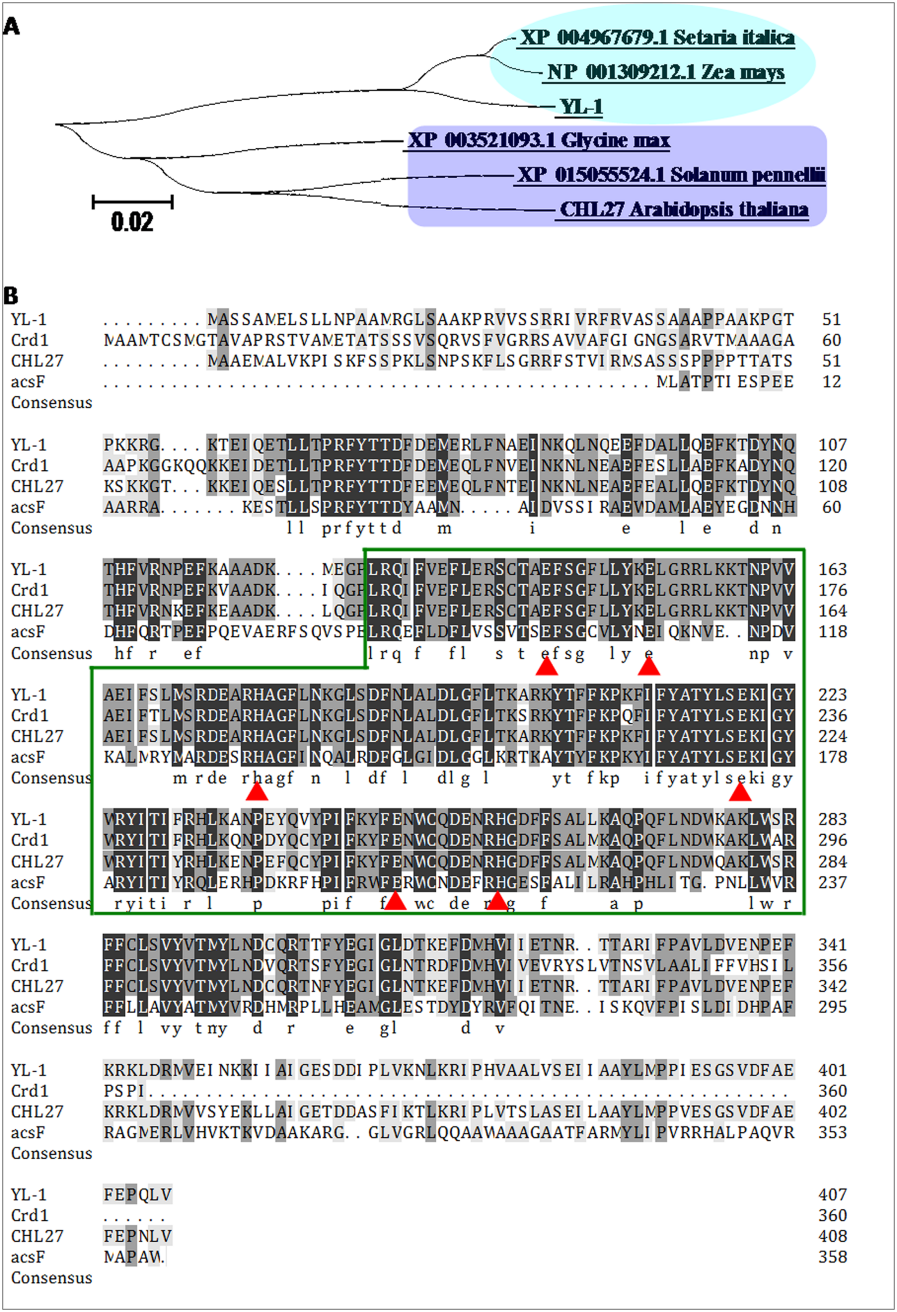
Phylogeny of the YL-1 protein family. A, phylogenetic tree representing alignment of YL-1 proteins. The oval-cyan box presents monocotyledons, and the rectangle-blue box means dicotyledons. The phylogenetic tree was constructed using the neighbor-joining algorithm. Bootstrap values were shown at each node. Bar Indicator of genetic distance based on branch length. B, Comparison of YL-1 protein sequences from both bacteria, green algae and plants. Black shading identical residues, gray shading similar residues. The green box presents the AcsF domain in different species, and the red-triangle box stands for the conserved residues of a diiron center (ion binding site) which is present in the AcsF domain.

The coding sequence of *YL-1* is 1227 bp and contains a 409-residue polypeptide with a molecular mass of approximately 47 kDa (http://rice.plantbiology.msu.edu). The *YL-1* sequence as a whole has homologs among other higher plants, particularly with sequences encoded by monocotyledonous species such as maize and *Setaria italica* (Fig 5A). Furthermore, *YL-1* encodes an MPMC with a AcsF domain (Fig 5B) which is highly conserved in bacteria, green algae and plants and often found in proteins which bind metal ion (Fig 5B).

### Transcription profiling of *YL-1* and subcellular localization of its product

**Fig 6:**
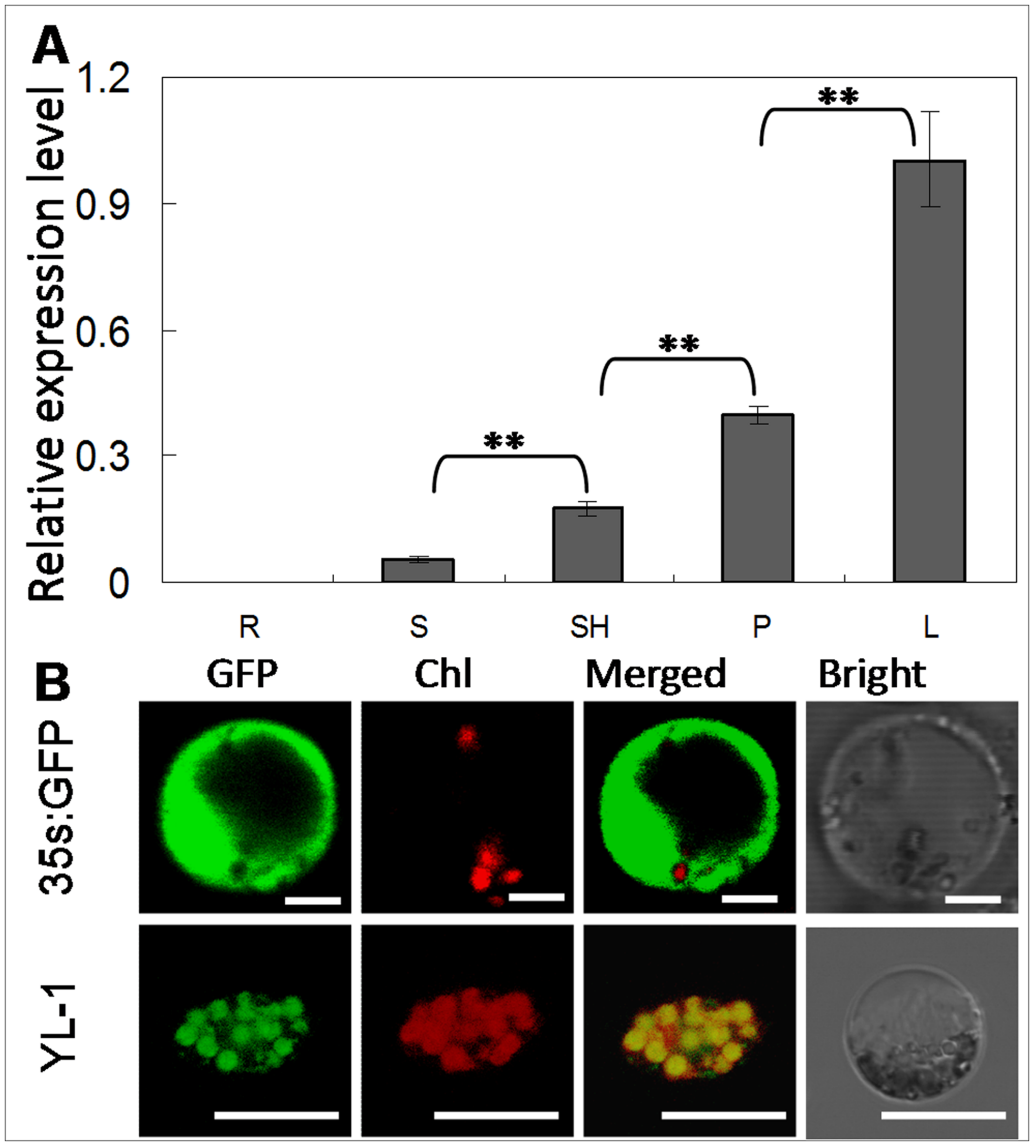
Expression pattern analysis and chloroplast localization of YL-1. **A**, Tissue-specific expression of *YL-1* at heading stage in wild-type. RNA was isolated from flag leaves (L), leaf sheaths (SH), culms (C), young panicles (P) and roots (R). **B**, GFP signal in tissue transformed with the empty GFP vector or YL-1-GFP fusion protein. Data in (**A**) are shown as means ± SD from 3 individual replicates. Bars = 2 μm in (**B**). The asterisks indicate statistical significance between the wild-type and the mutants, as determined by the Student’s *t*–test (* *P* < 0.05; ** *P* < 0.01).

Given that *yl-1* encodes a key enzyme subunit for chlorophyll biosynthesis, we determined its transcription levels in leaf photosynthetic tissues. When grown in the Hangzhou fields, *YL-1* were transcribed in the leaf, leaf sheath, culm, young panicle, and root. The expression profile of *YL-1* in these various tissues was investigated by qRT-PCR and the results showed that *YL-1* is constitutively expressed in the examined tissues, with higher levels in leaves, young panicles and leaf sheaths (Fig 6A), which corresponds to its protein function in chlorenchyma.

To identify the subcellular localization of the YL-1 protein, a transient expression system was conducted by delivering a *35S*::*YL-1*:*GFP* construct into rice protoplasts cells. The result showed that the GFP signal was localized in chloroplasts, while the signal pattern of *35S*::*GFP* control plastid was distributed over the whole cell (Fig 6B).

### The *yl-1* affects mRNA accumulation levels of Chl biosynthesis process

**Fig 7:**
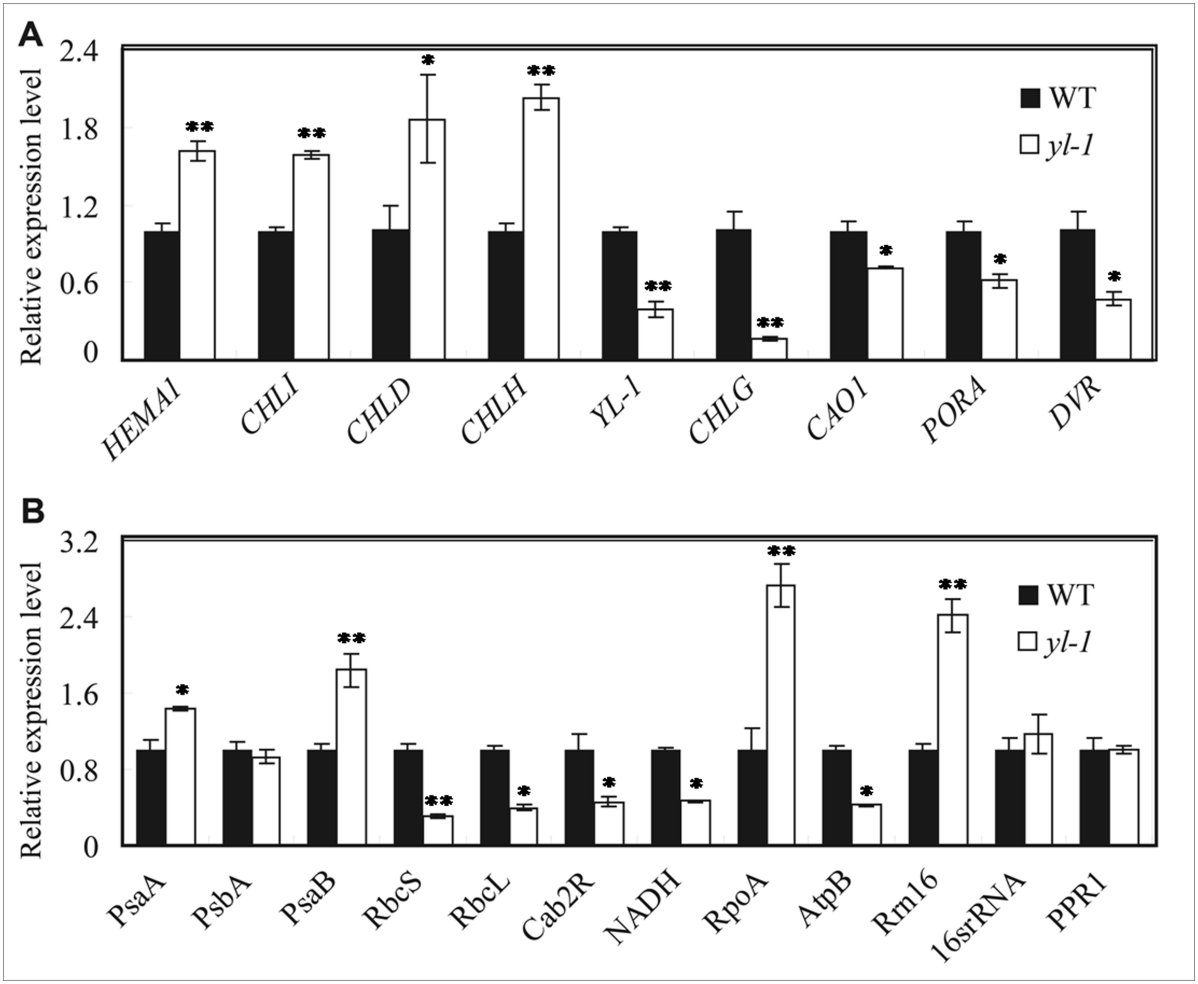
Transcriptional changes of chlorophyll biosynthesis-related genes and chloroplast development -associated genes between wild-type and *yl-1* mutant. **A**, Expression levels of genes related to Chlorophyll biosynthesis. **B**, Expression levels of Photosynthesis- and chloroplast development-related genes. Data are shown as means ± SD from 3 individual replicates. The asterisks indicate statistical significance between the wild-type and *yl-1*, as determined by the Student’s *t*–test (* *P* < 0.05; ** *P* < 0.01).

MPEC catalyzes the conversion of MPME to DVpchlide, which plays an important role in the central process of Chl biosynthesis. Therefore, the expression of genes involved in Chl biosynthesis were examined by qRT-PCR in both *yl-1* and wild-type plants. The genes, upstream of Chl biosynthesis, such as *glutaml-tRNA reductase (HEMA1)*, *Mg chelatase D subunit (CHLD)*, *Mg chelatase H subunit (CHLH)* and *Mg chelatase I subunit (CHLI)* were all up-regulated in the *yl-1* mutant, compared to wild-type (Fig 7A). On the other hand, the genes down-stream of Chl biosynthesis, such as *chlorophyllide a oxygenase (CHLG*, *YGL1)*, *chlorophyllide a oxygenase 1* (*CAO1*), *protochlorophyllide A (PORA)* and *divinylreductase (DVR)* were significantly decreased in the *yl-1* mutant (Fig 7A). In addition, we investigated the expression levels of genes involved in chloroplast development and photosynthesis. The chloroplast development depends on the regulation of two major RNA polymerase: nuclear-encoded RNA polymerase (NEP) and plastid-encoded RNA polymerase (PEP). In *yl-1* plants we found significant changes in the expression of PEP-dependent genes, such as the subunit of photosystem I gene *psaA*, a core component of Photosystem II gene *psbA*, the photosynthesis apparatus Rubisco large subunit gene *rbcL* and Rubisco small subunit gene *rbcS*. The NEP-dependent genes, such as *rpoA* and *rrn16* which highly expressed at early stages of chloroplast development were significantly increased in *yl-1* (Fig 7B). Moreover, transcription of photosynthesis-related genes *Oscab2R* and *NADH* were remarkably down-regulated in the *yl-1* mutant (Fig 7B). These data suggest that abnormal expressions of these chloroplast associated genes may be responsible for the chlorisis phenotype of *yl-1*.

## Discussion

Chl is extremely important in high-photosynthesis and plays an essential role in harvesting light energy and converting it to chemical energy. Generally, Chl deficiency affects the chloroplast development and photosynthesis, leading to abnormal leaf morphology including virescent (v), stripe (st), albino, chlorina, zebra, and yellow-variegated [36, 37]. Some of these Chl-deficient mutants are seedling-specific and gradually become normal during later growth stages, and some can affect the plants for the entire life cycle. The *yss1* rice mutant shows striated leaves at the seedling stage, particularly in leaf 3, but produces normal wild-type leaves later [38]. While, the *vyl* mutant develops decreased Chl levels throughout the whole developmental stages [39]. Here, we identified a novel Chl-deficient mutant *yl-1* with yellow leaf and yellow panicle during its entire growth duration, which is compatible with its reduced chlorophyll content, abnormal chloroplast morphology, and reduced photochemical efficiency.

Certain Chl-deficient mutants show abnormal phenotypes in response to temperature changes. Some are sensitive to lower temperature, including *v*_*1*_, *v*_*2*_ and *wlp1*, these all show albino phenotypes at a restrictive low temperature [40–42]. Others often show yellowing, stripe or completely albinism when subjected to high temperature. Although the *yl-1* mutant presents chlorisis under a range of temperature conditions, at higher temperature, there is a more serious yellow leaf phenotype with much more defects in Chl synthesis, which indicated high temperature may aggravate the effect of mutant phenotype of *yl-1*, due to *YL-1* could likely involved in the temperature-regulated chlorophyll biosynthesis.

**Fig 8:**
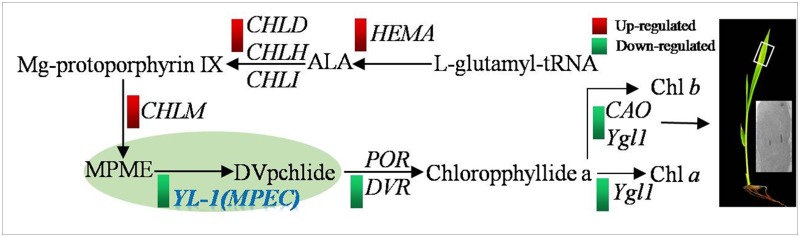
Model showing mutation of *YL-1* caused the abnormal Chl biosynthesis and leaf development in the mutant plant.

Map-based cloning suggests that *YL-1* encodes an enzyme, MPMC, essential for during Chl synthesis, which catalyzes the conversion of MgPME to Dvpchlide. Chl synthesis is a complex process starting with the formation of ALA and proceeding to the synthesis of Chl a and Chl b. In this study, we found extensive up-regulation of *HEMA*, which encodes the first enzyme that controls the formation of ALA, and of three genes encoding subunits of magnesium-ion chelating enzyme (*CHLD*, *CHLH*, *CHLI*), which play roles upstream of Chl synthesis. In addition, expression levels of down-stream genes including *YGL1*, *CAO1*, *PORA* and *DVR* were all significantly reduced. The results indicates that normal function of *YL-1* is essential for Chl biosynthesis, where it plays central role. In addition, we found that transcription level of *YL-1*was dramatically reduced in the *yl-1* mutant, which may interfere with its enzyme activity (Fig 8). Recently, *ygl8*, a novel gene was reported in rice, which also shows defects in Chl synthesis and chloroplast development with chlorisis phenotype [31]. Taken these evidences, we concluded that *YL-1* may play a pivotal function during Chl synthesis (Fig 8).

The subcellular localization of YL-1 shows it to be a chloroplast-located protein. Meanwhile, *Arabidopsis CHL27* also encodes a chloroplastic protein which is distributed in the inner-envelope and thylokoid membranes[28, 29]. Moerover, MPMC has been reported to exist in chloroplasts as a membrane-interacting multi-subunit enzyme containing at least one soluble and two membrane bound components [26]. In this study, RNA-seq analysis showed that expression of many membrane-associated genes, including plastid membrane-associated genes, was altered in the *yl-1* mutant, compared to wild-type, including the chloroplast outer envelope 24 kDa protein, and the membrane-associated 30 kDa protein (S2 Fig and S2 Table). It has been reported that the synthesis of ALA and Mg-protoporphyrin IX take place in chloroplast stroma, while, the subsequent processes of Chl synthesis including the conversion of MPME to Dvpchlide, and Chl *a*/*b* synthesis, are mostly catalyzed by membrane bound enzymes such as chlorophyllide a oxygenase, divinyl reductase and Chl synthase, which are located in the thylakoid membrane. In the *yl-1* mutant, loose thylakoid membranes and less dense grana stacks were observed, which indicated that the mutation of *YL-1* results in structure damage to thylakoid membranes. Furthermore, in *Arabidopsis*, CHL27, PORB, PORC and Geranylgeranyl reductase (GGR/CHLP) were identified as components of a FLU-CHL27-POR complex in chloroplast membranes, which plays important roles in Chl biosynthesis [30]. Thus, YL-1 has been demonstrated to be essential for chloroplast membrane stability in rice, even though there are no putative transmembrane domains in the YL-1 or CHL27 proteins.

There are many reports of rice leaf color mutants, including *v*_*1*_, *v*_*2*_, *wlp1* [40–42] and *yss1* [38]. Most of them were used as morphological marker applied in roguing of hybrid rice seed production and parents seed multiplication. However, many of the leaves color mutant showed their color change phenotype only under particular temperature regimes or specific development periods. Therefore they are not useful for identifying variation. In addition if roguing in hybrid rice seed production or parents seed multiplication was not carried out in a timely manner, these morphological markers would be useless and cause huge losses. For *yl-1*, the yellow leaf and panicle phenotype was stable and lasted the whole growth duration, which makes it suitable for roguing during the plant breeding processes. Furthermore, compared to wild-type, *yl-1* showed excellent agronomic traits of increased tiller number and reduced plant height, which is desirable in hybrid rice, especially in male sterile line rice.

In conclusion, we identified YL-1, function as MPEC subunit in rice, which might be essential for Chl biosynthesis and chloroplast development in rice. And the yellow leaf and panicle phenotype, together with improved agronomic traits of *yl-1* would be useful in male sterile line rice.

## Supporting information

S1 Fig The major agronomic traits of wild-type and *yl-1* mutant. (TIF)

S2 Fig RNA-seq analysis of the *yl-1* mutant and wild-type. (TIF)

S1 Table Genetic analysis of the *YL-1* mutant gene. (DOCX)

S2 Table The expressions of selected DEGs associated with Chl biosynthesis, chloroplast development, and photosynthesis. (DOCX)

S3 Table Primer sequences used in this study. (DOCX)
