# Supplementary figures and images for: Yellow-Leaf 1 encodes a magnesium-protoporphyrin IX monomethyl ester cyclase, involved in chlorophyll biosynthesis in rice (Oryza sativa L.)
Source: PLoS One. 2017 May 30;12(5):e0177989. doi: 10.1371/journal.pone.0177989 (PMC5448749; doi:10.1371/journal.pone.0177989)

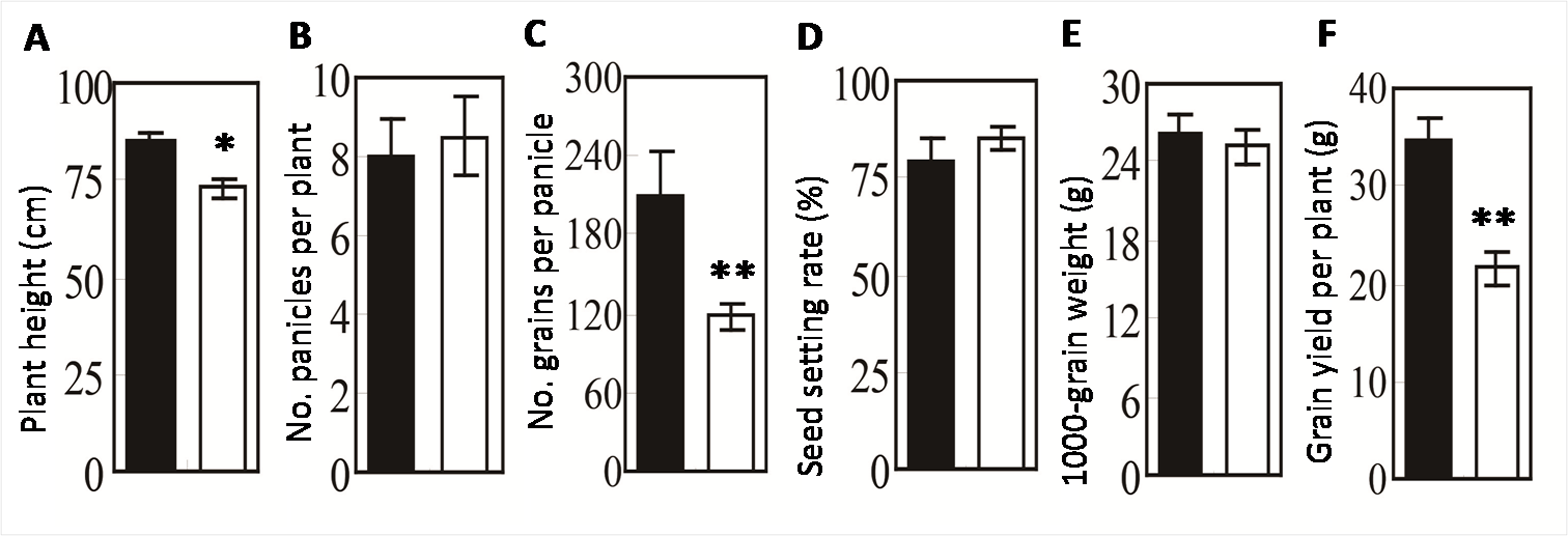

Supplement: S1 Fig — (TIF) [file pone.0177989.s001.tif]
