# Supplementary material for: Yellow-Leaf 1 encodes a magnesium-protoporphyrin IX monomethyl ester cyclase, involved in chlorophyll biosynthesis in rice (Oryza sativa L.)
Source: PLoS One. 2017 May 30;12(5):e0177989. doi: 10.1371/journal.pone.0177989 (PMC5448749; doi:10.1371/journal.pone.0177989)
Supplement: S1 Table — (DOCX) [file pone.0177989.s003.docx]

**S1 Table.** **Genetic analysis of the *YL-1* mutant gene.**

The segregation behavior in each of the two derived F_2_ populations was consistent with the Mendelian monogenic ratio of three wild type phenotype to one chlorisis.

| **F_2_ population** | **Total** | **Green individuals** | **Yellow individuals** | **Green /yellow**  **individuals** | **χ^2^_c_** | **χ^2^_0.05_** |
| --- | --- | --- | --- | --- | --- | --- |
| *yl-1*/D50 | 433 | 322 | 101 | 3.19 | 0.04 | 3.84 |
| *yl-1*/dular | 272 | 201 | 62 | 3.24 | 0.01 | 3.84 |
| *yl-1*/02428 | 289 | 206 | 71 | 2.90 | 0.81 | 3.84 |
