# Supplementary material for: Yellow-Leaf 1 encodes a magnesium-protoporphyrin IX monomethyl ester cyclase, involved in chlorophyll biosynthesis in rice (Oryza sativa L.)
Source: PLoS One. 2017 May 30;12(5):e0177989. doi: 10.1371/journal.pone.0177989 (PMC5448749; doi:10.1371/journal.pone.0177989)
Supplement: S2 Table — (DOCX) [file pone.0177989.s004.docx]

**S2 Table. The expressions of selected DEGs associated with Chl biosynthesis,**

| **Accession** | **Name** | ***yl-1*_fpkm** | **WT_fpkm** | **log2(fold_change)** | **Up or down regulated** | **p_value** |
| --- | --- | --- | --- | --- | --- | --- |
| LOC_Os03g21890 | *HEME2* | 82.31 | 65.58 | 0.327788475191026 | Up | 0.00 |
| LOC_Os04g52130 | *HEMF1* | 33.04 | 14.56 | 1.18231769331576 | Up | 0.00 |
| LOC_Os01g18320 | *HEMG1* | 8.71 | 3.12 | 1.4793593460093 | Up | 0.00 |
| LOC_Os03g20700 | *CHLH* | 233.38 | 229.04 | 0.027081004642111 | Up | 0.02 |
| LOC_Os03g36540 | *OsChlI* | 31.26 | 20.67 | 0.596359296729452 | Up | 0.00 |
| LOC_Os06g04150 | *CHLM* | 44.72 | 43.63 | 0.0354973724221864 | Up | 0.00 |
| LOC_Os10g41780 | *OsCAO1* | 86.89 | 114.85 | -0.402497607883383 | Down | 0.00 |
| LOC_Os03g22780 | *OsDVR* | 67.17 | 99.58 | -0.568065662155685 | Down | 0.00 |
| LOC_Os10g35370 | *PORB* | 61.46 | 88.91 | -0.53275951070753 | Down | 0.00 |
| LOC_Os05g28200 | *YGL1* | 10.34 | 11.82 | -0.192700887115954 | Down | 0.02 |
| LOC_Os01g17170 | *YL-1* | 670.81 | 1124.37 | -0.74513646363824 | Down | 0.00 |
| LOC_Os02g32520 | *OsClpD1* | 121.29 | 62.09 | 0.966082044524744 | Up | 0.02 |
| LOC_Os05g40180 | *OsSTN8* | 11.17 | 22.17 | -0.989404726026062 | Down | 0.03 |
| LOC_Os12g23200 | *PsaL* | 2211.77 | 2578.01 | -0.221056491072869 | Down | 0.00 |
| LOC_Os01g31690 | *PsbO* | 3824.18 | 5206.43 | -0.445144038102527 | Down | 0.04 |
| LOC_Os01g43070 | *PsbP* | 4.01 | 6.04 | -0.591518422660203 | Down | 0.04 |
| LOC_Os01g71190 | *Psb28* | 386.74 | 485.32 | -0.327557405002053 | Down | 0.00 |
| LOC_Os12g08770 | *PSI-N* | 3192.43 | 3789.36 | -0.247299220011164 | Down | 0.02 |
| LOC_Os07g05365 | *PsbR* | 7.70 | 0.01 | 9.58828551051523 | Up | 0.04 |
| LOC_Os01g14410 | *Chl a-b binding protein* | 243.22 | 1180.83 | -2.27944378179191 | Down | 0.00 |
| LOC_Os10g21352 | *Rps19* | 69.94 | 118.57 | -0.761676597520665 | Down | 0.00 |
| LOC_Os02g15900 | *ASL2* | 20.89 | 41.63 | -0.9950323419201 | Down | 0.02 |
| LOC_Os01g17150 | *Rpl5* | 147.62 | 212.32 | -0.524392011312345 | Down | 0.04 |
| LOC_Os01g57958 | *Rpl16* | 5.53 | 10.44 | -0.916129911642346 | Down | 0.00 |

**chloroplast development, and photosynthesis.**
