# Supplementary material for: Yellow-Leaf 1 encodes a magnesium-protoporphyrin IX monomethyl ester cyclase, involved in chlorophyll biosynthesis in rice (Oryza sativa L.)
Source: PLoS One. 2017 May 30;12(5):e0177989. doi: 10.1371/journal.pone.0177989 (PMC5448749; doi:10.1371/journal.pone.0177989)
Supplement: S3 Table — (DOCX) [file pone.0177989.s005.docx]

**S3 Table.** **Primer sequences used in this study.**

| **Marker** | | **Forward sequence (5’-3’)** | **Reverse sequence (5’-3’)** |
| --- | --- | --- | --- |
| **For fine mapping** | | | |
| RM3746 | | AAATGGGCTTCCTCCTCTTC | CAGCCTTGATCGGAAGTAGC |
| RM493 | | TAGCTCCAACAGGATCGACC | GTACGTAAACGCGGAAGGTG |
| RM10390 | | GCAACGTTACGTCTTGGCATGG | CCTCTCGCGTCTCTCTCAACG |
| RM10489 | | CTCGTTGAGAGGAAGGCCAACG | TGCCACGCAGTAGAATAAGCTCTCG |
| RM10551 | | AGCTTCTCCTCCTCCTCTTCTCG | CGCCAATCTCAAATCGAAATCC |
| RM10605 | | CTTCGTCGCAGCCCAAGAGAGG | GGCAGTACGGGCACTTGATCTCC |
| RM10644 | | TCCGGCAAATATAGTCCAAACG | CAACGACGACGACATGTATAGGC |
| RM10656 | | GCTGAGAGCTGATTAATCGGAAGC | CGCCACTAGCTAGCACACACC |
| RM10622 | | CGCGGTTAATGTCATCTGATTGG | CCATACTTCGAGATCCAAGACTGACC |
| Indel4 | | ACAATCAACTCTAAATGGATAACTAC | GCCTATGCTTTGCTACGGA |
| Indel8 | | CCATTAGGTCTTTCTTGTGCC | GTCCGTAGGTGGTGTTGG |
| **For quantitative PCR** | |  |  |
| *Ubq* | | GCTCCGTGGCGGTATCAT | CGGCAGTTGACAGCCCTAG |
| *HEMA1* | | CACCAGTCTGAATCATAT | CTACCACTTCTCTAATCC |
| *CHLD* | | GGAAAGAGAGGGCATTAG | CAATACGATCAAGTAAGTGTT |
| *CHLI* | | AGTAACCTTGGTGCTGTG | AATCCATCAACATTCAACTCTG |
| *CHLH* | CTATACATTCGCCACACT | | TATCACACAACTCCCAAG |
| *YGL1* | TGGACAGTTGAAGATGTT | | GAATAGGACGGTAAGGTT |
| *PORA* | ATCACCAAGGGCTACGTCTC | | GAGTTGTTGTTCCAGCTCCA |
| *psaA* | GAGATACCACTTCCTCAT | | ACTAAGAAATTCTGCGTATT |
| *psbA* | AAGTTTCTCTGATGGTATG | | ATAGCACTGAATAGGGAA |
| *rbcL* | GTTGAAAGGGATAAGTTGA | | AATGGTTGTGAGTTTACG |
| *CAO1* | GATCCATACCCGATCGACAT | | CGAGAGACATCCGGTAGAGC |
| *YL-1* | AAGCCCAAGTTCATCTTCTACGC | | TCATCCTGGCACCAGTTCTCAA |
| *PPR1* | CTAAGACCGAATGACAAATGC | | GCACTGCCAACAAGAATACC |
| *rbcS* | TCCGCTGAGTTTTGGCTATTT | | GGACTTGAGCCCTGGAAGG |
| *DVR* | CGAGCCCAGGTTCATCAAGGTGC | | CCTCCCGATCTTGCCGAACTCC |
| *Cab2R* | TGTTCTCCATGTTCGGCTTCT | | GCTACGGTCCCCACTTCACT |
| *NADH* | GGGCAAATCGGATATGTAAT | | CGAAGAAACCTGCTAGTGGA |
| *RpoA* | GTGGAAGTGTGTTGAATCAA | | TCTCTCTTGATCCGTAACTC |
| *AtpB* | TTTGGTTTCGATGTGCA | | TATGGTCTAATTCCGAGCGGT |
| *Rrn16* | AACAATGACGGTATCTGAGGAATAAG | | CTGGGATTTGACGGCGGACT |
| *16srRNA* | CCGTTGGTGTTCTTTCCGAT | | TTCAAGTCCGCCGTCAAATC |
| **For vector construction** |  |  |  |
| OE- *YL-1* | TTACTTCTGCACTAGGTACCTCTCGCCACCTTATCTCATC | | GAATTCCCGGGGATCCTAACCACC  CATCATCTCACC |
| *YL-1-*GFP | GCCCAGATCAACTAGTATGGCCTCCTCCGCCATGGAG | | TGCTCACCATGGATCCTTAATTTTG  TAAACAAGAAAA |
